# Genetic diversity and population structure among native, naturalized, and invasive populations of the common yellow monkeyflower, *Mimulus guttatus* (Phrymaceae)

**DOI:** 10.1002/ece3.9596

**Published:** 2023-04-07

**Authors:** Elizabeth A. Zimmer, Jason A. Berg, Michele R. Dudash

**Affiliations:** ^1^ Department of Botany and Laboratories of Analytical Biology, National Museum of Natural History Smithsonian Institution Washington District of Columbia USA; ^2^ Department of Biological Sciences University of Maryland College Park Maryland USA; ^3^ Department of Natural Resource Management South Dakota State University Brookings South Dakota USA

**Keywords:** introduced populations, microsatellite profiling, *Mimulus guttatus*, native populations

## Abstract

An ongoing controversy in invasion biology is the prevalence of colonizing plant populations that are able to establish and spread, while maintaining limited amounts of genetic variation. Invasive populations can be established through several routes including from a single source or from multiple introductions. The aim of this study was to examine genetic diversity in populations of *Mimulus guttatus* in the United Kingdom, where the species is considered invasive, and compare this diversity to that in native populations on the west coast of North America. Additionally, we looked at diversity in non‐native populations that have not yet become invasive (naturalized populations) in eastern North America. We investigated population structure among populations in these three regions and attempted to uncover the sources for populations that have established in the naturalized and invasive regions. We found that genetic diversity was, on average, relatively high in populations from the invasive UK region and comparable to native populations. Contrastingly, two naturalized *M. guttatus* populations were low in both genetic and genotypic diversity, indicating a history of asexual reproduction and self‐fertilization. A third naturalized population was found to be a polyploid *Mimulus* hybrid of unknown origin. Our results demonstrate that *M. guttatus* has likely achieved colonization success outside of its native western North America distribution by a variety of establishment pathways, including those with genetic and demographic benefits resulting from multiple introductions in the UK, reproductive assurance through selfing, and asexual reproduction in eastern North America, and possible polyploidization in one Canadian population.

## Graphical abstract

We investigated the genetic footprint of *Mimulus guttatus* native, naturalized, and invasive populations in the Western US, Northeastern America, and the United Kingdom, respectively. Naturalized populations had low genetic variation relative to native and invasive ones.

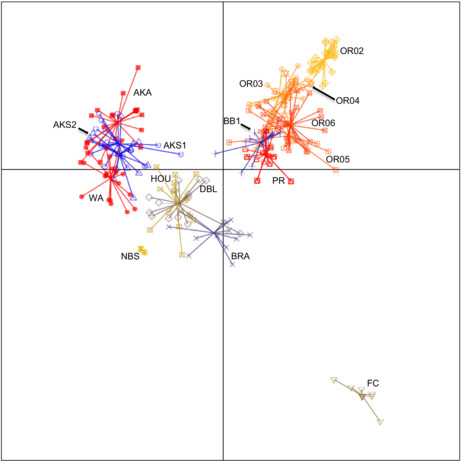

## 1 INTRODUCTION

Establishment by non‐native plant species around the globe is a well‐documented occurrence, and the probability of successfully colonizing a novel location outside of the species' native range depends on many variables. Factors that can restrict the establishment and spread of introduced plant populations include low genetic diversity and Allee effects associated with founder events, insufficient propagule pressure in the form of a single or few introductions, maladaptation to novel environmental pressures, or some combination of these (Lee, 2002; Lockwood et al., 2013; Richardson et al., 2000; Szczecinska et al., 2016; Williamson & Fitter, 1996). However, when a nascent plant population is able to overcome the influence of a novel suite of environmental pressures, the formation of establishment pathways can lead to a stage in the invasion process called naturalization.

According to Richardson et al. (2000), naturalized populations are those non‐native plant populations that maintain sufficient population size by sexual reproduction or asexual vegetative proliferation, so that the probability of extinction due to environmental stochasticity is low. Naturalization is often considered an intermediate stage prior to a population becoming invasive, representing a lag phase of slow population growth as it deals with deficiencies inherent to a novel population's demographics or to maladaptation (Aikio et al., 2010; Frappier et al., 2004; Murren et al., 2009; Richardson & Pyšek, 2012). The naturalization stage is considered in many theoretical models of invasion a critical point in determining whether a non‐native population goes extinct, remains cryptic and benign, or alternatively adapts and spreads aggressively into new locations (Catford et al., 2009; Guo et al., 2018; Richardson & Rejmánek, 2011). Despite the importance of the naturalization stage in characterizing the progression from casual colonization to impactful invasion in predictive models, few empirical studies include genetic diversity data from naturalized populations to compare with native and invasive populations (Pysek et al., 2008). This gap in our understanding is largely due to the difficulty in locating and recognizing naturalized populations prior to their becoming invasive (Aikio et al., 2010).

The important transition from naturalization to invasion is often dictated by the genetic constitution of the plant population, which is in turn governed by the mode of reproduction and mating system of the plant species in question (Garcia‐Ramos & Rodriguez, 2002; Kinlan & Hastings, 2005). Invasive plant species display extensive variation regarding the importance of sexual versus asexual reproduction, and the degree to which sexual reproduction relies on outcrossing (i.e., mating between unrelated individuals) versus self‐fertilization (Barrett et al., 2008). The mode of reproduction determines establishment and invasion potential because it influences population genetic parameters such as the amount of additive genetic variation, effective population size, and partitioning of genetic diversity within and among populations (Barrett, 1998; Eckert et al., 2010; Lopez‐Villalobos & Eckert, 2018; Vogler & Kalisz, 2001).

Invasion processes have resulted in transitions to higher rates of self‐fertilization in mixed‐mating plants (e.g., selfing and asexual reproduction) in the introduced range (Barrett, 2011; Barrett et al., 2008; Clements & Ditommaso, 2011). The study of self‐fertilization and clonality and their role in facilitating colonization of non‐native locations goes back decades to “Baker's Law” (Baker, 1955; Stebbins, 1957). Baker suggested that self‐compatible species should theoretically be more successful colonizers following long‐distance dispersal compared with obligate outcrossing species, in part because the former would need only one individual to establish a naturalized population (Kolar & Lodge, 2001). Asexual reproduction can also contribute to an introduced plant population's establishment and persistence in heterogenic environments, such as riparian ecosystems or roadside seeps (Cushman & Gaffney, 2010) that often results in a single or few genotypes expanding in an area, evidenced by the most successful aquatic plant invader, the water hyacinth, *Eichhornia crassipes* (Zhang et al., 2010).

While a single introduction, followed by some form of uniparental reproduction, has been shown to be a successful strategy to become naturalized, a more common scenario appears to be by multiple introductions of plant propagules followed by at least occasional outcrossing (Dlugosch & Parker, 2008; Wilson et al., 2009). When outcrossing is the primary mode of reproduction, additive genetic variation within the population increases compared with populations that rely on selfing that results in a 50% decrease in heterozygosity after each generation (Carr & Dudash, 2003; Charlesworth & Charlesworth, 1987). By enhancing genetic variation, outcrossing enables an incipient population to respond and adapt more quickly to the changes in environmental conditions common during invasion (Barrett et al., 2008; Charlesworth, 2003; Lynch & Walsh, 1998). Outcrossing can also result in interspecific hybridization among closely related species that have recently come into contact, and examples of allopolyploid species such as *Tragopogon mirus* (Soltis et al., 2004), *Senecio cambrensis* (Abbott & Lowe, 2004), and a *Spartina* hybrid cross between the *S. foliosa* and *S. alterniflora* (Ainouche et al., 2009; Ayres et al., 2004) have been described. Hybridization among sister taxa, whether they share a common ploidy level or not (e.g., diploid × tetraploid = triploid hybrid; Vallejo‐Marin & Lye, 2013), may stimulate invasiveness through heterosis or recombination (Baack & Rieseberg, 2007).

In this study, we use molecular data to examine the genetic diversity and structure among a range of native and non‐native populations of diverse origins and residence times. Specifically, we compare two naturalized eastern North American populations of the mixed‐mating plant species, *Mimulus guttatus* D.C. (Phrymaceae), a nearby naturalized population comprised of a heretofore‐undescribed hybrid *Mimulus* taxon, three non‐native *M. guttatus* populations in the United Kingdom, where the species is considered invasive, and native populations that occur across a large span of the species' home range in western North America. Our goal was to address the following questions: (1) How does genetic and genotypic diversity in non‐native populations (i.e., naturalized and invasive populations) compare with diversity in native populations? We predicted that populations in the invasive region will have similar levels of genetic diversity as native populations due to the species' history of multiple introductions as an ornamental plant (Truscott et al., 2008); (2) Which native location is most likely the source for non‐native *M. guttatus* populations? We predicted that non‐native *M. guttatus* populations in the UK are derived from populations on the northern edge of the native distribution based on prior evidence (Puzey & Vallejo‐Marín, 2014). There has been no prior investigation regarding the source region for naturalized populations on the east coast of North America, and we aim to shed light on the origin of these non‐native populations.

## 2 MATERIALS AND METHODS

### 2.1 Study species

*Mimulus guttatus* (2*n* = 2*x* = 28), or common monkeyflower, is a herbaceous species native to the west coast of North America, found from Mexico to Alaska (Carr et al., 1997; Dudash et al., 1997; Kelly & Arathi, 2003; Lowry et al., 2008; Wu et al., 2008). In the United Kingdom (UK), *M. guttatus* is considered a harmful invasive that was intentionally introduced as a horticultural species approximately 200 years ago (Truscott et al., 2008; van Kleunen & Fischer, 2008). Recently, naturalized populations in New York State and New Brunswick, Canada, have received attention (Murren et al., 2009). Little is known of the evolutionary history of these naturalized populations, but they are thought to have established at least 50 years ago. Native populations of *M. guttatus* are described as mixed‐mating and exhibit wide variation in outcrossing rates (Dudash & Carr, 1998; Ritland & Ganders, 1987). To our knowledge, outcrossing rates in naturalized and invasive populations have not been estimated. *Mimulus guttatus* is also capable of asexual reproduction via fragmentation and stolons (Grant, 1924; Truscott et al., 2006; Vickery, 1959). *Mimulus guttatus* has become a model system in studies of ecological and evolutionary genomics because of its broad phenotypic and genetic diversity (Dudash et al., 2005; Wu et al., 2008). In its native range, *M. guttatus* populations can be found as either annuals or perennials, and this difference in life history depends on water availability (van Kleunen, 2007; Lowry et al., 2008; M. R. Dudash & C. J. Murren, unpublished data). For this study, we only sampled from perennial native populations because all known non‐native populations are perennial.

### 2.2 Sampling sites

To compare genetic diversity and population structure among *M. guttatus* populations in the native, naturalized, and invasive ranges, we conducted fieldwork in 2012 and 2013 (Table 1 includes estimates of population size). Our sampling of native populations along the west coast of North America covered a large latitudinal transect (~5150 km) from Point Reyes, CA to Seward, AK. The route to survey 11 native perennial populations was based on records obtained from colleagues (B. Blackman & D. Lowry, personal communication) and local contacts. In the naturalized region on the east coast of North America, two of the three populations sampled were previously studied by Murren et al. (2009) and located in Fly Creek, New York and Springfield, New Brunswick, Canada. Local botanists provided the location of a third population, near Bass River, New Brunswick (NBBR). Initially, the NBBR population was thought to be comprised of *M. guttatus* individuals. However, following assessment of genotyping data, chromosome counts, and morphological traits (e.g., low pollen viability and reduced seed set in the greenhouse), it was apparent that the NBBR population was comprised of polyploid hybrid *Mimulus* individuals. These individuals likely represent a polyploid (2*n* = 3*x* = 42–46; J. A. Berg, unpublished data) with *M. guttatus* constituting at least one of the parental taxa. It has been shown that *M. guttatus* readily hybridizes with closely related *Mimulus* species to form allopolyploids (Clausen et al., 1950; Vallejo‐Marin, 2012; Vickery, 1978), including the triploid hybrid *M. × robertsii* Silverside (2*n* = 3*x* = 44–46) formed by *M. guttatus* and a South American species, *M. luteus*, which is also found throughout the UK (Vallejo‐Marin & Lye, 2013). The second parent taxon of the NBBR hybrid population is likely a tetraploid *Mimulus* species, but its identification was beyond the scope of this study. Here, we utilize the NBBR population in the surveys of genetic diversity, but do not include it in the structure analyses due to the difficulties that arise in combining polyploid and diploid data in these assessments.

**TABLE 1:** Location and approximate population size of 11 *Mimulus guttatus* populations from the native region, two from the naturalized region, and three from the invasive region) used for genetic analysis.

	Code	Latitude	Longitude	Approximate no. of individuals in population
Native region populations				
Seward, AK (1)	AKS1	60.07021	−149.28102	>1000
Seward, AK (2)	AKS2	60.12142	−149.25660	>200
Anchorage, AK	AKA	63.33945	−148.49102	>1000
Shelton, WA	WA	47.23089	−123.08862	>1000
Oswald State Park, OR	OR06	45.45797	−123.58105	>1000
Cloverdale, OR	OR05	45.14347	−123.58188	>100
Heceta Head, OR	OR04	44.08163	−124.07605	>500
Otter Point, OR	OR03	42.27994	−124.25329	>1000
Humbug Mt. State Park, OR	OR02	42.43072	−124.27879	>500
Bodega Bay, CA	BB1	38.31701	−123.07117	>1000
Point Reyes, CA	PR	37.997989	−122.995067	>1000
Naturalized region				
Bass River, New Brunswick, Canada (polyploid hybrid)	NBBR	46.32904	−65.06621	>500
Fly Creek, NY	FC	42.44391	−74.58212	>1000
Springfield, New Brunswick, Canada	NBS	45.41486	−65.49202	>500
Invasive region				
Brampton, Norfolk, UK	BRA	52.7681	−1.27985	>100
Dunblane, Perthshire, UK	DBL	56.18861	−3.96608	>300
Houghton Lodge, Hampton, UK	HOU	51.09699	−1.5084	>100

*Note*: A third naturalized population included in the genetic and genotypic diversity analyses was a putative polyploid *Mimulus* hybrid population (NBBR).

For each of the 14 native and naturalized populations, we randomly sampled fruits and leaf tissue from 30 to 50 individual plants that were >1 m apart to increase the chances of sampling multiple genotypes. Leaf tissue was immediately stored in silica gel until DNA extraction. For the three *M. guttatus* populations in the invasive region in the UK, we obtained field‐collected seed from a colleague, Mario Vallejo‐Marin, at the University of Stirling in Scotland. This seed was then grown at the University of Maryland (UMD) greenhouse, and when seedlings were ~6 cm tall, leaf tissue was collected from 20 individuals per population and stored in silica gel. Following DNA extraction and genotyping (see below), the sample size was reduced to 14 individuals for each of the UK populations due to poor‐quality DNA in some samples.

### 2.3 Genetic markers

To genotype the 17 populations from the native, naturalized, and invasive regions, we used 12 codominant markers (Table 2) including six microsatellite loci previously used to genotype North American and British *Mimulus* populations (Kelly & Willis, 1998; Vallejo‐Marin & Lye, 2013), and six markers revealing length polymorphisms in introns of single‐copy nuclear genes in *M. guttatus*, *M. × robertsii*, and *M. luteus* (Fishman & Willis, 2005; Lowry et al., 2008; Vallejo‐Marin & Lye, 2013). These intron‐length polymorphisms, or MgSTS (*Mimulus guttatus* sequence‐tagged sites) are suitable for genotyping based on a selection strategy of Vallejo‐Marín et al. (2011). These markers have been shown to be variable in samples of *M. guttatus*, its close relative, the tetraploid *M. luteus*, and the triploid hybrid produced by them, *M. × robertsii* Silverside and are suitable for multiplexing (Vallejo‐Marin & Lye, 2013).

**TABLE 2:** Number of alleles and observed heterozygosity for 16 *Mimulus guttatus* populations and one polyploid *Mimulus* hybrid population (NBBR) at six microsatellite loci (*AAT*) and six intron‐based length polymorphism markers (*MgSTS*).

Locus	Approx. size range (bp)	*M. guttatus*		Polyploid hybrid		*Both taxa*
		Total no. of alleles	*H*_O_	Total no. of alleles	*H*_O_	Total no. of alleles
AAT217	177–195	6	0.129	4	1.00	10
AAT225	113–127	9	0.103	2	0	11
AAT230	179–210	21	0.252	2	1.00	23
AAT240	94–106	5	0.100	3	1.00	8
AAT267	117–131	4	0.139	2	0	6
AAT278	127–135	4	0.090	3	0.96	7
MgSTS84	209–231	8	0.213	2	0	10
MgSTS234	272–321	6	0.190	2	0	8
MgSTS321	290–303	9	0.313	4	0	13
MgSTS430	238–269	9	0.277	3	0.96	12
MgSTS681	338–366	10	0.332	3	0.92	13
MgSTS685	242–251	9	0.359	2	0	11
Average		8.33	0.223	2.667	0.487	11.0
SD		4.52	0.091	0.778	0.509	4.411
Total		100		32		132

*Note*: Forward primers for the first six markers on the list (AAT217–AAT278) were labeled with FAM dye, and the remaining six markers (MgSTS84–MgSTS685) had forward primers labeled with HEX dye. Number of individuals analyzed per taxon (number of populations): *M. guttatus*: 310 (16); triploid *Mimulus* hybrid population: 26 (1); for each locus, only individuals amplifying for at least one allele were used in calculations of the parameters shown.

### 2.4 DNA extraction PCR amplification

To extract DNA from leaf tissue, a modified CTAB protocol (Doyle & Doyle, 1990) was employed on an AutoGenprep 965/960 instrument (AutoGen) using the Plant DNA Extraction Kit AGP965/960, following the manufacturer's protocol. DNAs were amplified for the 12 loci in sets of two multiplexed reactions (Table 2) using a 2× Qiagen Type‐It Microsatellite PCR kit (Qiagen), 2 μM of each of the fluorescent forward primers labeled with either FAM or HEX dyes and 2 μM of each reverse primer and 5–50 ng of template DNA. PCR cycles consisted of a denaturing step of 5 min at 95°C, followed by 30 cycles of 95°C for 30 s, 55°C for 180 s and 72°C for 30 s, and a final elongation step of 60°C for 30 min. We examined success of the PCR amplifications in a 1.5% agarose 1× sodium hydroxide‐boric acid buffer electrophoresis gel (Brody & Kern, 2004). PCR products were diluted in nuclease‐free water (dilutions ranged from 1:10 to 1:50), and 1 μl of each dilution was added to 9 μl of HiDi formamide with 1 μl ROX standard (DeWoody et al., 2004). Samples were heated to 95°C for 6 min, cooled to 4°C for 6 min, and loaded onto an ABI 3730xl automated capillary sequencer with a 50 cm, 96 channel array containing POP‐7 polymer for fragment analysis at the Laboratories of Analytical Biology (LAB) of the Smithsonian National Museum of Natural History.

### 2.5 Genetic analyses

We performed allele binning and analyzed raw peak sizes from fluorescent fragment profiles using GeneMapper v5.0 software (Applied Biosystems), which allows calling of multiple peaks per locus. A random sample of 10% of individuals was reassayed and rescored to check consistency. For the polyploid individuals at the NBBR site, determining conventional genetic diversity parameters based on allele frequency (e.g., expected heterozygosity) is problematic because of the difficulty in identifying alleles in partial heterozygotes. Therefore, to assess allelic diversity in each population and between regions, we calculated the following statistics (Sampson & Byrne, 2012): the total number of alleles across all loci (*A*); the average number of alleles per locus in each population (*A'*); the average number of alleles per locus in an individual (*H′*); the proportion of observed heterozygotes, averaged over all loci (*H*_O_); and the number of private alleles (*P*). Welch's two‐sample *t*‐tests were used to test for significant differences between regions for these statistics (the NBBR population containing polyploid individuals was excluded from *t*‐tests). For the diploid *M. guttatus* populations, we used GenAlEx 6.5 to calculate expected heterozygosity and deviations from Hardy–Weinberg equilibrium (HWE). To determine pairwise genetic differences between individuals within each population, we used the method developed for microsatellite data by Bruvo et al. (2004) in the R package *poppr* (Kamvar et al., 2014). The distance measure of Bruvo et al. (2004) is similar to band‐sharing indices and is appropriate for relative distance comparison among intraspecific individuals of different ploidy levels and takes into account stepwise mutational processes.

We used *poppr* for multilocus genotype (MLG) assignment, to determine expected proportion of MLGs from the total number of individuals sampled (*R*) using a rarefaction method to account for sample size (Hurlbert, 1971) and to calculate the complement of Simpson's diversity index *D* (Simpson, 1949).

### 2.6 Population genetic structure

We used four complementary analyses to investigate population structure among the 16 *M. guttatus* populations in the native, naturalized, and invasive regions: (1) a hierarchical analysis of molecular variance (AMOVA; Excoffier et al., 1992); (2) a discriminant analysis of principal components (DAPC); (3) clustering of the 16 *M. guttatus* populations based on Nei's genetic distance (Nei, 1978) visualized using a dendrogram; and (4) Mantel tests to examine the correlation between geographic and genetic distance in order to detect cases of isolation by distance. The putative polyploid *Mimulus* hybrid population from the naturalized region (NBBR population) was left out of the analyses of population structure because it was the only polyploid population found. This omission allowed us to focus on the structure among the remaining diploid *M. guttatus* populations.

First, we conducted a hierarchical AMOVA in *poppr* to estimate variance and distribution of diversity within and among regions, populations and individuals within populations. The significance of variance components calculated for all levels was tested with 1000 permutations.

The second analysis, DAPC, is a multivariate method to identify clusters comprised of genetically similar individuals (Jombart et al., 2010). DAPC uses principle components derived from principle components analysis (PCA) as variables to optimize between‐group variation and minimize within‐group variation in order to separate individuals into predefined groups (Jombart et al., 2010). The method uses a *k*‐means clustering algorithm to analyze any number of potential clusters (*k*'s) in a sequential manner. The optimal *k* should correspond with the lowest Bayesian Information Criterion (BIC) score. DAPC has been suggested as an alternative to other Bayesian clustering methods such as STRUCTURE (Pritchard et al., 2000) because it does not require a population genetic model to identify clusters. Therefore, DAPCs are suitable for analyzing complex genetic data sets such as those that may not adhere to the assumption of random mating within populations (e.g., clonality or self‐fertilization). The DAPC analysis was conducted in the R package *adegenet* 1.3‐1 (Jombart & Ahmed, 2011). Prior to DAPC analysis, we used the *adegenet* function *clonecorrect* to account for clonality in the data set. Next, to find the optimal number of clusters, we used *k*‐means clustering of principal components using the function *find. clusters* in *adegenet*. The function *xvalDapc* was used to cross‐validate the number of principal components used in the analysis.

To complement the DAPC analysis and help resolve structure among the 16 *M. guttatus* populations, we constructed a dendrogram based on Nei's genetic distance (Nei, 1978). Data were bootstrapped in the R package *poppr* using the *aboot* function from a sample of 1000 bootstrapped trees. The function *clonecorrect* was applied to the data prior to bootstrapping to account for clonality within populations.

Finally, to examine the relationship between geographic location and genetic differentiation and the possible existence of isolation by distance (IBD), three separate Mantel tests were performed with the *ade4* package in R, using the function *mantel.rtest*. The output of each of the three tests was based on a Monte Carlo method using 1000 replicates. The first test included all 16 *M. guttatus* populations from the three regions, the second test included the 11 native *M. guttatus* populations only, and the third test included the 5 non‐native populations from the naturalized and invasive regions. The measure of pairwise genetic distance between populations used was Jost's *D*, which measures the fraction of allelic variation among populations (Jost, 2008). Jost's *D* will equal unity at complete differentiation, and zero with no differentiation between populations. Geographic distance between each pair of populations was recorded as the shortest distance between populations and measured in kilometers. The function *clonecorrect* was applied to the data prior to each of the Mantel tests.

## 3 RESULTS

### 3.1 Genetic and genotypic diversity within populations and between regions

Individual genotypes consisted of either one or two peaks per locus in the 16 *M. guttatus* populations and ranged from one to three peaks in the putative polyploid hybrid population NBBR. Markers deviating from Hardy–Weinberg equilibrium (HWE) were found in each of the 11 native and three invasive *M. guttatus* populations. The two naturalized *M. guttatus* populations, NBS (New Brunswick, Canada) and FC (New York), were monomorphic for 11 and seven loci, respectively (the polyploid NBBR population was left out of structure analyses). All loci that were not monomorphic in these populations deviated from HWE. The total number of alleles amplified per locus (*A'*) ranged from 6 at locus AAT267 to 23 at locus AAT230, when both the *M. guttatus* and the putative polyploid taxa were considered (Table 2). In total, 100 different alleles (*A*) were amplified in the 16 *M. guttatus* populations and 32 alleles in the putative polyploid (NBBR) population for the 12 microsatellite and intron‐length polymorphic markers. Of the 32 alleles from the polyploid hybrid population, six (19%) were found exclusively in that population.

Levels of intrapopulation genetic diversity (*A*, *A'*, *H′*, *H*_O_, *P*; Table 3) in the native and invasive regions were generally higher than those in the naturalized region, but not significantly so (Welch's two‐sample *t*‐tests; *p* > .05). The total number of alleles (*A*; Table 3) in the native and invasive regions (28.27 ± 4.24 and 30.67 ± 7.67, respectively) was nearly twice that found in the two naturalized *M. guttatus* populations, NBS and FC (15.50 ± 3.54). The largest number of alleles was found in the putative polyploid NBBR population in the naturalized region (32), while the fewest was found in the naturalized *M. guttatus* population NBS (13; Table 3). Populations in the native and invasive regions also averaged more alleles per locus (*A'*; Table 3) than those in the naturalized region (native: 2.35 ± 0.34; invasive: 2.55 ± 0.64; naturalized: 1.30 ± 0.28; *p* < .06 for both comparisons, naturalized vs. native and naturalized vs. invasive). The putative polyploid NBBR population had the greatest number of alleles per locus (2.7). Average observed heterozygosity in the native and invasive region was similar (0.22 ± 0.05 and 0.26 ± 0.06, respectively; Table 3) and twice that of the two naturalized *M. guttatus* populations (0.12 ± 0.16), while heterozygosity in the NBBR population was relatively high (0.49), as expected for a population consisting of polyploid individuals (Husband & Schemske, 1997). The naturalized FC population had the highest number of private alleles (five) among non‐native *M. guttatus* populations. The other naturalized population, NBS, had zero private alleles. The populations from the invasive region averaged 2.3 private alleles (SD + 0.58; Table 3).

**TABLE 3:** Measures of genotypic and genetic diversity of 16 *Mimulus guttatus* populations and 1 polyploid *Mimulus* hybrid population (NBBR) sampled from three regions, native (western North America), naturalized (eastern North America), and invasive (United Kingdom).

Population	*N*	*G*	*R*	*D*	*A*	*A'*	*H′*	*H*_O_	*P*	Mean pairwise difference (±SD)
Native region										
AKS1	18	17	0.97	0.94	25	2.1	1.19	0.20	0	0.164
AKS2	16	16	1.0	0.94	28	2.3	1.24	0.25	2	0.171
AKA	22	20	0.95	0.95	24	2.0	1.18	0.18	1	0.115
WA	20	20	1.0	0.95	36	3.0	1.27	0.28	4	0.209
OR06	27	24	0.94	0.95	24	2.0	1.15	0.17	0	0.109
OR05	12	12	1.0	0.92	22	1.8	1.16	0.16	2	0.167
OR04	15	15	1.0	0.93	29	2.4	1.18	0.17	6	0.171
OR03	13	13	1.0	0.92	31	2.6	1.24	0.24	3	0.202
OR02	28	27	0.98	0.96	30	2.5	1.15	0.16	3	0.146
BB1	17	17	1.0	0.94	33	2.75	1.29	0.30	2	0.186
PR	29	16	0.78	0.92	29	2.4	1.26	0.26	2	0.158
Mean ± SD	19.7 (±6.0)	17.9 (±4.5)	0.97 (±0.07)	0.94 (±0.01)	28.27 (±4.24)	2.35 (±0.34)	1.21 (±0.05)	0.22 (±0.05)	2.27 (±1.74)	0.163 (SE = ±0.009)
Total Native region	217	197			311				25	
Naturalized region										
NBBR	26	6	0.28	0.34	32	2.7	1.64	0.49	6	0.012
FC	21	7	0.37	0.48	18	1.5	1.30	0.23	5	0.028
NBS	30	2	0.17	0.44	13	1.1	1.00	0	0	0.019
Mean ± SD (FC and NBS only)	25.50 (±6.36)	4.50 (±3.54)	0.27 (±0.14)	0.46 (±0.03)	15.5 (±3.54)	1.30 (±0.28)	1.15 (±0.21)	0.12 (±0.16)	2.50 (±3.54)	0.024 (SE = ±0.005)
Total Naturalized region	77	15			63				5	
Invasive region										
BRA	14	14	1.0	0.93	24	2.0	1.32	0.32	2	0.189
DBL	14	14	1.0	0.93	39	3.25	1.25	0.25	3	0.249
HOU	14	14	1.0	0.93	29	2.4	1.20	0.20	2	0.236
Mean ± SD	14 (±0)	14 (±0)	1.0 (±0)	0.93 (±0)	30.67 (±7.64)	2.55 (±0.64)	1.26 (±0.06)	0.26 (±0.06)	2.33 (±0.58)	0.225 (SE = ±0.018)
Total Invasive region	42	42			92				7	

*Note*: *N*, number of sampled individuals; *G*, number of multilocus genotypes; *R*, expected proportion of multilocus genotypes from total number of sampled individuals using rarefaction to account for sample size; *D*, complement of Simpson's index of diversity; *A*, total number of alleles seen over all loci; *A*′, average number of alleles per locus in the population; *H′*, average number of alleles per locus per individual; *H*_O_, proportion of individuals with heterozygous genotype (averaged over loci); *P*, number of private alleles. Pairwise genetic distance based on method developed by Bruvo et al. (2004).

The mean pairwise genetic difference between individuals (*H′*; Table 3) in each population was lowest, on average, in the naturalized region (0.024 ± 0.005), greater within native populations (0.163 ± 0.009), and greatest in the invasive region (0.225 ± 0.018). Mean pairwise genetic distance in the NBBR population was the lowest of all populations and similar to distances in the other two naturalized populations (0.012). The mean number of unique multilocus genotypes (MLGs; Table 3) was 17.9 ± 4.5 in the native populations versus 14.0 ± 0 in populations in the invasive region and 4.5 ± 3.54 in naturalized populations. Only six MLGs represented the 26 NBBR individuals sampled. The mean proportion of MLGs among the number of individuals sampled, following rarefaction to account for differences in sample size (*R*; Table 3), was 1.0 in the invasive region (all sampled individuals within populations represented a unique MLG), 0.97 ± 0.07 in the native region, and 0.27 ± 0.14 in the naturalized region. The proportion of MLGs in the NBBR population (0.28) was similar to the two *M. guttatus* naturalized populations. There was very little difference between the native and invasive regions' mean complement of Simpson's diversity (*D*; Table 3) indices (0.94 ± 0.01 and 0.93 ± 0, respectively), and both indices were more than twice that found in the naturalized region (0.46 ± 0.03) and nearly threefold larger than the *D* calculated for NBBR (0.34).

### 3.2 Population genetic structure

An AMOVA (Table 4) with a three‐level hierarchy (three regions, populations, and individuals) indicated that 46.33% of diversity was maintained across populations and 37.71% among individuals within populations (Φ_Population, Region_ = 0.55, *p* < .001; Φ_Individuals, Total_ = 0.62, *p* < .001; Table 4), while only 15.97% of diversity was maintained among regions (Φ_Region, Total_ = 0.16, *p* < .001).

**TABLE 4:** Analysis of molecular variance (AMOVA) for 16 *Mimulus guttatus* populations and 1 polyploid *Mimulus* hybrid population, following correction for identical MLGs (clone correction) within each population.

Grouping (16 *M. guttatus* populations)	Source of variation	df	Variance components	Variance (%)
Regions/populations/individuals	Among regions	2	1.38*	15.97
	Among populations within regions	13	4.01*	46.33
	Within populations	232	3.26*	37.71
	Total	247	8.65	100.00

*Note*: Stars indicate significant (*p* < .001) structure at a given hierarchical level.

**FIGURE 1:**
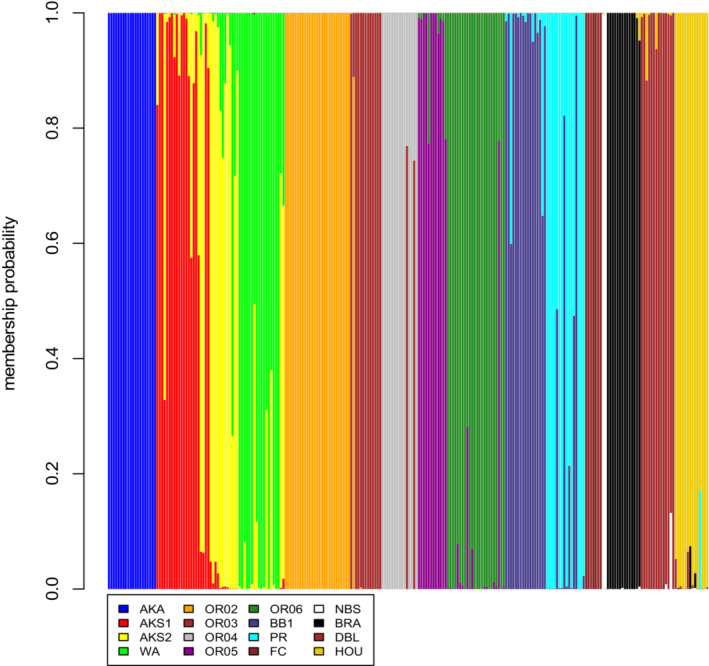
Cluster plot of DAPC for 248 multilocus genotypes (MLGs) from 16 diploid *Mimulus guttatus* populations. Illustrates the probability of membership for each MLG to 16 genetic clusters identified in the analysis (*k* = 16). Populations AKA through PR are from the native region; FC and NBS are from the naturalized region; BRA, DBL, and HOU are from the invasive region. Population codes are provided in Table 1.

The discriminant analysis of principal components (DAPC) showed that the k‐means clustering separated the data set (248 MLGs representing 310 diploid *M. guttatus* individuals from 16 populations) into 16 clusters (Figure 1), indicating that each sampled population was genetically distinct from one another. To address the possibility of overfitting in the DAPC, which can occur if too many principal components are withheld in the model (Jombart et al., 2010), we ran the analysis several times using a range of principal components from 5 to 50. Each time, the optimal number of clusters was 16 based on the Bayesian information criterion.

**FIGURE 2:**
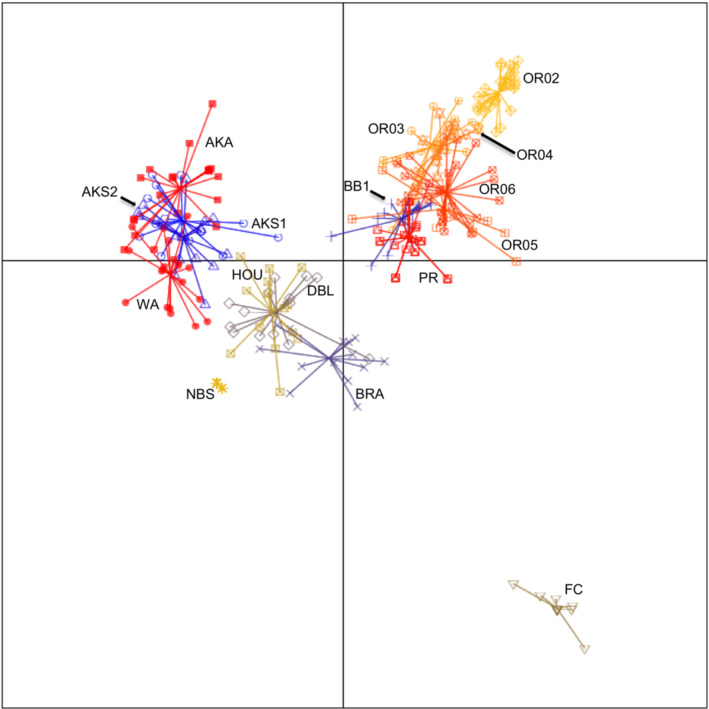
Scatterplot of DAPC of the first two principal components discriminating 16 diploid *Mimulus guttatus* populations. Points are 248 sampled multilocus genotypes. Lines and shapes represent population membership. The optimal number of clusters (*k*) identified in the DAPC analysis was 16, matching the number of sampled populations (11 from the native region, 2 from the naturalized region, and 3 from the invasive region; population codes are found in Table 1).

When plotted along the first two principle components used for the DAPC, the 16 populations sorted into four distinct groups (Figure 2). The first group consisted of four of the 11 native populations and included the three Alaskan populations and the Washington State population. The second group included the remaining seven native populations, specifically the five Oregon populations and the two California populations. The third group consisted of the three populations from the invasive region in the UK, along with the naturalized population from Springfield, New Brunswick (NBS). The fourth group consisted of a single population, from Fly Creek, New York (FC) in the naturalized region on the east coast of North America.

**FIGURE 3:**
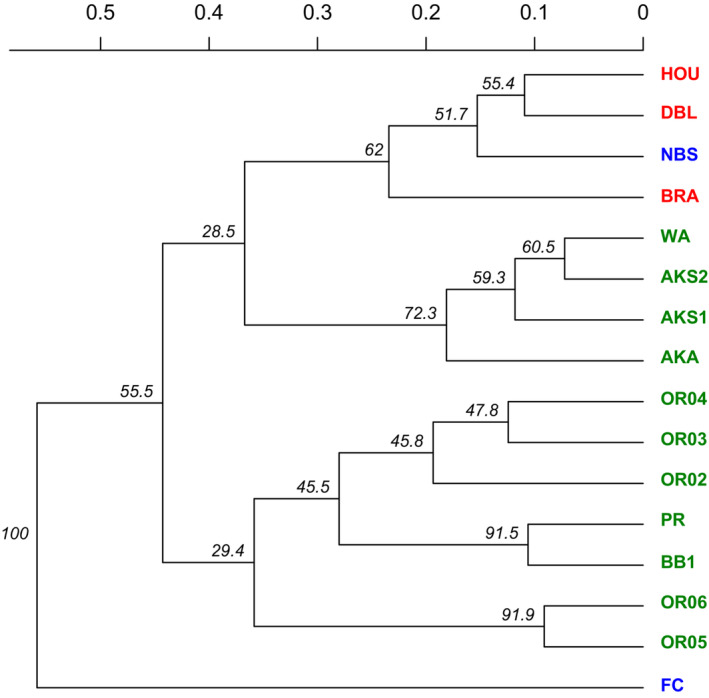
Dendrogram based on Nei's genetic distance (Nei, 1978) detailing genetic structure among the 16 diploid *Mimulus guttatus* populations. Populations in red are from the invasive region in the UK; blue from the naturalized region on the east coast of North America; and green are native populations from the west coast of North America. Scale bar shows relative genetic difference between populations, and numbers at nodes are bootstrap values for a sampling of 1000 trees.

A dendrogram based on Nei's genetic distance (Nei, 1978) provided complementary evidence of the genetic structure among the 16 *M. guttatus* populations (Figure 3). Each of the 1000 trees sampled for bootstrapping showed that the naturalized FC population was genetically distinct from the other 15 populations, which corresponded to this population's distinct placement determined by the principle components (Figure 2). Also, the dendrogram showed 62% support for a clade consisting of the three populations from the invasive region in the UK and the *M. guttatus* population from Springfield, New Brunswick, in the naturalized region (NBS). The three native populations from Alaska, along with the population from Washington state, formed a clade within the larger grouping of native populations (72% support). This AK/WA clade was also shown as being the most closely related native group to the invasive UK/NBS clade; however, support for this relationship was low (28.5%).

To examine the relationship between genetic and geographic distance, we performed three Mantel tests (*r*_m_; Table 5): The first test included all 16 *M. guttatus* populations and showed a positive correlation between genetic and geographic distance (*r*_m_ = .33; *p* < .01), which indicates a classic pattern of isolation by distance. The second test included only the 11 native populations and also showed a positive correlation with geographic distance (*r*_m_ = .36; *p* < .01). The third test included the five non‐native populations (excluding the putative polyploid NBBR population) and, contrary to the previous tests, showed little correlation between genetic and geographic distance (*r*_m_ = 29; *p* > .1).

**TABLE 5:** Three separate Mantel tests to test for isolation by distance among (a) all 16 *Mimulus guttatus* populations, native, naturalized, and invasive; (b) the 11 native populations only; (c) the five naturalized and invasive populations only (the polyploid NBBR population was excluded from Mantel tests). Jost's *D* was used as the genetic distance.

Mantel test	*r*_m_
(a) 16 native and non‐native populations	.33*
(b) 11 native populations	.36*
(c) five non‐native populations	.29

*Note*: A significant correlation (*r*_m_) indicates isolation by distance. An asterisk indicates a significant results at the *p* < .01 level.

## 4 DISCUSSION

Understanding the role of genetic variation in plant invasions is a primary focus in modern ecology, and reconciling the paradox that exists when plant populations with low genetic diversity are able to establish in non‐native locations is a goal for researchers interested in managing these populations (Dlugosch & Parker, 2008; Lee, 2002; Moran & Alexander, 2014). To shed light on this issue, our study examined the genetic diversity and structure of naturalized populations of a potentially invasive plant species, *Mimulus guttatus*, and compared this diversity to populations in the native and invasive regions. Using molecular data from microsatellite and intron‐length polymorphic markers, our study revealed several major findings: (1) The naturalized *M. guttatus* populations in eastern North America had low genetic diversity compared with populations in the native and invasive regions, so the occurrence of only a few multilocus genotypes in naturalized populations suggests a population bottleneck and persistence due to a selfing/asexual reproduction; (2) we found some evidence for the northern edge of the native distribution representing the source location for populations in the invasive region, as well as data suggesting a close relationship between the invasive UK populations and one of the naturalized populations on the east coast of North America; (3) one naturalized population in New Brunswick, Canada, was identified as a polyploid species of *Mimulus*. Overall, we have provided evidence for multiple pathways of establishment for the non‐native populations of *M. guttatus*. Later, we discuss our results in more detail and consider their relevance to broader questions in invasion genetics.

### 4.1 Genetic and genotypic diversity in native, naturalized, and invasive regions

Our comparison of genetic diversity revealed that in the two naturalized *M. guttatus* populations, NBS and FC, diversity was substantially lower, on average, than that found in populations located in the native region. While these differences were not statistically significant at the *p* = .05 level (due in part to the low level of detectable genetic variability in the naturalized region), we found that the average total number of alleles, number of alleles per locus, and observed heterozygosity in native populations were nearly twice that found in the two naturalized populations (Table 3). Genetic diversity in the NBS (Springfield, New Brunswick, Canada) was effectively nonexistent, with 11 homozygous loci and only one locus (MgSTS84) segregating for two alleles. This lack of genetic diversity, coupled with the presence of only two multilocus genotypes (MLGs) in the 30 individuals sampled (i.e., low genotypic diversity), suggests that the NBS population is the product of a colonizing cohort of *M. guttatus* individuals that were subjected to founder effects following introduction. This population could be the result of a single introduction of a few propagules, followed by a reliance on asexual reproduction and/or self‐mating to persist. Alternatively, the NBS population could have resulted from multiple introductions that were subjected to environmental filters that allowed only a few genotypes to establish.

There are examples of invasive plants that have become invasive while maintaining low genetic diversity relative to native populations; however, most of these examples reproduce apomictically or rely solely on some form of uniparental reproduction (Bakker et al., 2009; Dlugosch & Parker, 2008; Fennell et al., 2010; Roux et al., 2011). Examples of mixed‐mating species becoming invasive despite low genetic diversity, as we found in the NBS population, are much less common. The results of Hagenblad et al. (2015) are one such example, in which invasive populations of the mixed‐mating species *Impatiens glandulifera* in Europe had much lower genetic diversity than native populations from India despite being introduced multiple times. The researchers concluded that phenotypic plasticity, a characteristic expressed in many introduced plant populations and thought to influence invasion success (Davidson et al., 2011; Murren & Dudash, 2012), might have played a large role in allowing populations with depauperate genetic diversity to establish and spread in novel environments far from the native region (Hagenblad et al., 2015). Murren and Dudash (2012) have also found evidence for increased expression of phenotypic plasticity in certain architectural traits in *M. guttatus* when grown in field sites in non‐native locations. Perhaps the two MLGs in the NBS population that we uncovered in this study were selected for certain adaptive plastic traits that suited them well following introduction into the remote location in Springfield, New Brunswick, Canada. Given the low genetic variation and genotypic diversity in the NBS population, a logical next step would be to examine these individuals for their capacity to express plasticity in novel, stressful environments. A companion greenhouse study was conducted to shed light on the role of phenotypic plasticity in response to abiotic conditions that naturally vary among native and non‐native populations across their distribution in this study.

The current status of the NBS population has been categorized here as naturalized and not invasive because it consists of only a few hundred individuals restricted to a small area of approximately 1500 square feet. Like many introduced plant populations, we cannot be sure of the introduction history of the NBS population. Knowing the potential for *M. guttatus* to become invasive, it remains to be seen if the NBS population will spread by overcoming barriers restricting it to its current location. By monitoring this naturalized population over the coming years, we could learn much about the importance of environmental, demographic, and genetic factors in restricting *M. guttatus* from rapid population growth.

The second naturalized *M. guttatus* population in this study, FC (Fly Creek, New York), was also relatively deficient in some measures of genetic diversity relative to native populations but overall its observed heterozygosity was similar to the average heterozygosity found in the native populations. The FC population may have a similar introduction history as the NBS population, but it is unlikely they originated from the same source population (see discussion of source populations below). Methods designed to detect a recent reduction in population size based on the principle of excess heterozygosity using microsatellites or other molecular markers (Beaumont, 1999; Cornuet & Luikart, 1996; Garza & Williamson, 2001) typically require larger sample sizes than the six MLGs representing FC in this study in order to obtain robust statistical results. Therefore, based on our findings, further sampling is warranted to determine whether the naturalized *M. guttatus* population located in Fly Creek, New York, may be the product of a recent bottleneck, especially given our estimate of its population size of greater than 1000 individuals.

In the three populations from the invasive region in the UK, genetic and genotypic diversity was similar to the native populations, supporting the evidence of multiple introductions in this region and suggesting that outcrossing is the prominent mode of reproduction. *Mimulus guttatus* was introduced into the UK repeatedly as a horticultural species (Truscott et al., 2008; van Kleunen & Fischer, 2008), and this intentional, repeated introduction of propagules has likely manifested in high genotypic diversity. Each of the successfully sampled individuals in the three populations, BRA, DBL, and HOU represented a unique MLG (*n* = 14 successful samples from an original total of 20 individuals for each invasive population). While few morphological, environmental, or demographic variables can be considered as universal facilitators across all plant invasions, propagule pressure has been found to be a common denominator explaining nearly all successful invasions for which there are historical records of introduction (Colautti et al., 2006). Based on the scope of our study, we cannot say definitively that the greater genotypic diversity found in *M. guttatus* populations from the invasive region compared with the naturalized region provides an ample explanation as to why some *M. guttatus* populations become invasive while others do not. However, our results can be used in combination with future environmental comparisons and evaluations of residence times (Pysek et al., 2009) to develop a clearer picture of what factors may promote invasion in *M. guttatus*.

### 4.2 Sources for non‐native populations and population structure

Our complementary analyses of population genetic structure of the 16 diploid *M. guttatus* populations suggest that there was little gene flow among populations within or between the three regions. An AMOVA showed that more variation in genetic diversity was maintained between populations than between regions (46.33% of the variance vs. 15.97%, respectively; Table 4), and the DAPC clustered genotypes into 16 distinct groups (Figure 1). Despite these results demonstrating definitive population groupings, there was evidence for admixture between the two Seward, Alaska populations (AKS1 & AKS2), between the Washington (WA) and AKS2, between Oregon populations, and between the two California populations (BB1 & PR). Taken with the results of the Mantel test that included only the native populations, we can conclude isolation by distance is resulting in differentiation among native populations but admixture may occur between neighboring sites. However, this isolation by distance interpretation is contradicted by admixture data between the WA and AKS2 sites, which are separated by more than 2000 km. Our leading hypothesis is that the urban WA population originated from propagules from a source near the remote Alaska sites, on the northern edge of *M. guttatus*' northern distribution. Genetic variation in the WA population is relatively high, and the occurrence of four private alleles leads us to believe that the population may be the product of multiple immigrations from other regions of the native range.

We found no strong association that would identify any of the 11 native *M. guttatus* populations as a source for any non‐native populations in our study, although there was slight evidence for the native Alaskan group being the most closely related to the group comprised of the three invasive UK populations and the NBS population (Figure 3). This would support a prior study that identified the northern edge of the native distribution as the potential source for introduced populations in the UK. Using genome resequencing data, Puzey and Vallejo‐Marín (2014) found that several non‐native *M. guttatus* populations in the UK (including the DBL and HOU populations sampled for this study) are derived from a region in the North Pacific, specifically in the Queen Charlotte Islands in British Columbia, Canada. Future “Next‐Gen” sequencing may provide greater resolution of the geographic source region compared with traditional SSR marker techniques regarding introduction histories and establishment pathways, and recent methods for analysis such as approximate Bayesian computation can allow the evaluation of different invasion scenarios (Cristescu, 2015).

As mentioned above, we found some support for the inclusion of the naturalized NBS population within the clade formed by the UK populations in the invasive region (Figure 3, bootstrap value = 52%). That NBS was more closely related to the UK populations than the other east coast population, FC, was also supported by the Mantel test that included only the five non‐native *M. guttatus* populations. Had NBS been less differentiated from its closest neighbor on the east coast compared with the populations in Europe, this test would have shown a correlation between genetic and geographic distance; however, this was not the case. This result is interesting because it may reveal that the NBS population originated from a source in the invasive region in Europe or from the same population that sourced the UK populations, rather than from a source in the native region of western North America. This scenario would represent what is known as a bridgehead effect, which occurs when non‐native populations become invasive and subsequently serve as the source for nascent populations in remote new territories (Lombaert et al., 2010). Theoretically, populations that have become invasive have already been subjected to selective filters in environments outside of the native region and thus should be well adapted to colonizing new locations. Thus, if certain genotypes are more successful colonizers, either because they express traits that allow for persistence during founder effects (e.g., low inbreeding depression and exploitation of resources following disturbance) or they fit Baker's description of the “general‐purpose genotype” by being more phenotypically plastic than other genotypes (Baker, 1974), then the bridgehead effect could act as an efficient process for choosing adaptive colonizers that can leapfrog into other territories. It is plausible that the source population for the NBS population in New Brunswick, Canada, is located in Europe, as the two continents maintain a robust trade in horticultural products. From 2013 to 2015, the EU exported over $9 billion in horticultural products to the US alone. It is possible that the founding propagules that colonized the NBS site arrived from Europe prior to the enforcement of current efforts such as the USDA Plant Protection and Quarantine program, enacted to restrict the import of potentially invasive plant species.

The naturalized Fly Creek (FC) population located in New York demonstrated high differentiation from the other populations, completely isolated from other groups by the first two principal components (Figure 2) and received 100% bootstrap support as its own clade on the dendrogram (Figure 3). There are several possible explanations for high differentiation in naturalized populations, including founder effects combined with genetic drift (Bossdorf et al., 2005; Roman & Darling, 2007). Low genetic variation in naturalized plants will limit evolution and restrict the species' progression to the invasion stage (Müller‐Schärer et al., 2004) unless the species harbors variation for plasticity (Davidson et al., 2011).

### 4.3 The occurrence of a *Mimulus* hybrid in the naturalized region

While conducting this study, we identified the presence of a heretofore‐unknown *Mimulus* hybrid species in eastern North America. Chromosome counts conducted revealed between 44 and 46 chromosomes (J. A. Berg, unpublished data), greater than the 28 chromosomes typically found in the diploid *M. guttatus* (Vickery, 1995) but lower than the 56 chromosomes expected in a tetraploid. The North American *M. guttatus* is known to form mostly sterile triploid hybrids in the UK with closely related sister taxa from geographically disparate regions, namely the tetraploid South American species *M. luteus* (2*n* = 4*x* = 60–62) and *M. cupreus* (2*n* = 4*x* = 62) (Stace, 2010). Perhaps most notable is the triploid hybrid *M. × robertsii* (2*n* = 3*x* = 44–46; Silverside, 1990) formed by *M. guttatus* and *M. luteus*, which escaped cultivation in the late 19th century and has established several naturalized populations in the UK (Preston et al., 2002; Vallejo‐Marin, 2012). The chromosome counts of the NBBR individuals were similar to those found in UK *M. robertsii* individuals (Vallejo‐Marin, 2012). Also, pollen viability in the greenhouse was low (Berg, personal observation), as might be expected from a triploid species. However, we cannot infer that the NBBR individuals represent a newly found population of *M. × robertsii* in North America without further genome‐wide analyses. On the east coast of North America, neither *M. cupreus* or *M. luteus* has been recorded. The other yellow‐flowered species that has been found is *M. moschatus*, a non‐native escape from garden plots (Pennell, 1935). *Mimulus moschatus* is a tetraploid (2*n* = 4*x* = 32), which makes it a candidate as the second parental taxon with *M. guttatus* that could produce the putative triploid population NBBR. However, the results from chromosome counts revealed higher counts than would be expected from an *M. guttatus × M. moschatus* hybrid, and they have also been reported as being incompatible (Vallejo‐Marin, 2012).

Because most triploid *Mimulus* hybrids in the UK have been found to be largely sterile (Vallejo‐Marin, 2012; Vallejo‐Marin & Lye, 2013), the NBBR population may represent a pathway to establishment in the naturalized region directed by uniparental asexual reproduction. Its sterility and the fact that few potential parent *Mimulus* species occur on the east coast of North America to propagate more hybrids means that additional colonization by this hybrid would have to come from emigrants from the present population or future escapees. More research is required to definitively identify this population/species and its ploidy level before we can make accurate assessments concerning its potential to progress from a naturalized population to one that may begin to spread and become invasive on the east coast of North America.

## 5 CONCLUSION

Our study of *M. guttatus* populations from native, naturalized, and invasive regions demonstrates that naturalized populations in eastern North America have low genetic and genotypic variation compared with native populations on the west coast of North America. It is likely that these two naturalized populations experienced founder effects and rely on uniparental reproduction, asexual reproduction, and/or selfing, to persist. A third naturalized population in New Brunswick, Canada, was identified as a polyploid *Mimulus* species and may demonstrate interspecific hybridization as a successful pathway to establishment in remote novel areas. Populations in the invasive region in the UK have similar genetic and genotypic diversity as the native populations, an expected result because of their historical record of multiple introductions. The invasive region may have also served as the source population for the naturalized population, NBS, providing a possible example of the bridgehead effect. More work is required to determine whether naturalized populations are restricted from becoming invasive because they lack genetic variation, or because they are limited by environmental factors. By continuing to monitor these naturalized populations, we can learn much about the invasion process while controlling their potential spread.

## AUTHOR CONTRIBUTIONS

**Jason A. Berg:** Conceptualization (equal); data curation (lead); formal analysis (equal); investigation (lead); methodology (equal); resources (equal); validation (equal); writing – original draft (lead); writing – review and editing (equal). **Michele R. Dudash:** Conceptualization (equal); data curation (supporting); formal analysis (equal); funding acquisition (supporting); investigation (lead); methodology (supporting); project administration (supporting); resources (equal); supervision (supporting); validation (equal); visualization (supporting); writing – review and editing (equal). **Elizabeth A. Zimmer:** Conceptualization (equal); data curation (equal); funding acquisition (lead); investigation (supporting); methodology (equal); project administration (equal); resources (supporting); software (supporting); supervision (equal); validation (supporting); visualization (supporting); writing – review and editing (equal).

## Data Availability

Microsatellite profile information is available at https://doi.org/10.5061/dryad.cvdncjt7c.
